# Pelvic congestion syndrome (PCS) as a pathology of postmenopausal women: a case report with literature review

**DOI:** 10.1186/s12905-021-01323-3

**Published:** 2021-04-27

**Authors:** Thomas Bartl, Florian Wolf, Christian Dadak

**Affiliations:** 1 Department of Obstetrics and Gynecology, Division of General Gynecology and Gynecologic Oncology, Medical University of Vienna, Waehringer Guertel 18-20, 1090 Vienna, Austria; 2 Department of Biomedical Imaging and Image-Guided Therapy, Division of Cardiovascular and Interventional Radiology, Medical University of Vienna, Waehringer Guertel 18-20, 1090 Vienna, Austria

**Keywords:** Pelvic congestion, Embolization, Postmenopausal, Pelvic pain, Interventional radiology, Case report

## Abstract

**Background:**

Due to the scarcity of adequately powered, randomized controlled trials and internationally standardized diagnostic criteria, evidence on the diagnosis and treatment of pelvic congestion syndrome (PCS) is limited. Earlier epidemiologic observations led to the attribution of PCS to the premenopausal state, and a remission of symptoms after menopause is frequently described a hallmark of the pathology. This concept has currently been challenged by radiological studies reporting a notable prevalence of ovarian venous congestion in adult female patients of advanced age. PCS as a pathology of postmenopausal women, however, has not been acknowledged by systematic research to date, impeding appropriate diagnostics and therapy for affected patients.

**Case presentation:**

A 69-year-old postmenopausal patient presented with newly diagnosed dilated and insufficient pelvic veins in combination with characteristic pain anamnesis, thereby fulfilling the diagnostic criteria of PCS. Interventional coil embolization of both ovarian veins as a standard treatment previously described for premenopausal patients was successfully performed, resulting in prompt alleviation of symptoms. The patient remained symptom-free at the 18-month follow-up visit.

**Conclusions:**

Given this first systematically documented case of a patient with postmenopausal symptomatic PCS in the light of recently published data on the prevalence of ovarian venous congestion in patients of advanced age, it may be assumed that PCS is not to be considered a pathology strictly limited to premenopausal state. Further clinical studies expanding the diagnostic scope beyond menopause may help to substantiate evidence and subsequently define standardized therapeutic approaches for affected postmenopausal patients.

## Background

Pelvic congestion syndrome (PCS) is a disease with heterogeneous clinical presentation. After exclusion of other causative pathologies, PCS is defined by a combination of uterine or ovarian varicose veins and chronic pelvic pain for more than six months [1]. The VEIN-TERM transatlantic interdisciplinary consensus document of the American Venous Forum (AVF) describes PCS as a chronic venous disease, comprising pelvic and post-coital pain, perineal heaviness, and acute urinary urge, caused by ovarian or pelvic venous reflux or obstruction. Vulvar or perineal varicoses may also be observed [2]. Premenopausal multiparae appear to be the most affected [3]. The exact prevalence of the disease, however, remains unclear due to the lack of recent, interdisciplinary diagnostic criteria. Available retrospective analyses report a significant proportion of premenopausal women to demonstrate varicose pelvic veins in the pelvic area, which appear, however, to be rarely symptomatic [4, 5]. It has been estimated that up to 40% of all cases of female chronic pelvic pain might be related to PCS [6].

The pathogenesis of PCS is poorly understood and is primarily based on the observation of symptom alleviation after interventional or surgical treatment of congested pelvic veins. The heterogeneity of symptoms, however, points to multifactorial processes that foster pelvic venous insufficiency. Dysfunctional venous valves, a vasodilatory effect of estrogen, and impaired involution after mechanical injury in late pregnancy have been proposed to cause primary PCS [6, 7]. Secondary PCS is characterized by anatomical obstruction, such as the venous compression in nutcracker or May-Thurner syndromes [3, 8]. Despite etiological differences, either type of PCS is similarly characterized by an increase in the volume of pelvic veins in combination with characteristic pain, which tends to worsen toward the end of the day or after long periods of standing due to orthostatic pressure. This observation is interpreted as a local release of pain and inflammation-mediating factors such as bradykinin or substance P as well as mechanical compression of local structures such as nerves [9, 10].

Radiological examination demonstrating insufficient, congested pelvic veins presents the second pillar of PCS diagnosis. Guidelines or consensus statements regarding the choice of radiological techniques or diagnostic cutoffs for pathologic venous diameters, however, are not available to date [11]. Special consideration must be given to the position of the patient during imaging, however, as supine positions may not represent maximum pelvic vein dilatation. To date, there is no evidence supporting primary surgical approaches if they are not indicated for other pathologies. In particular, any clinical benefit of laparoscopy has to be addressed critically as the combination of patient position and increased intraperitoneal pressure is likely to conceal congested veins [12].

Considering the lack of standardized diagnostic criteria and high rate of under-diagnosis, correct anamnesis remains the main pillar of correct PCS diagnosis [13]. PCS, however, has been primarily associated with the premenopausal state and postmenopausal remission of symptoms has been termed a hallmark of PCS [6, 14]. This perception is challenged by recent, sporadic observations of newly diagnosed symptomatic postmenopausal patients with pelvic or vulvovaginal varicose veins, suggesting that limiting diagnostic criteria to premenopausal patients may prevent a subset of patients to access available therapeutic options [15, 16]. To address this potential diagnostic gap, we thereby present the first systematic report on a symptomatic postmenopausal PCS patient who experienced a full alleviation of symptoms after receiving the standard-of-care usually applied to respective premenopausal patients.

## Case presentation

**Fig. 1:**
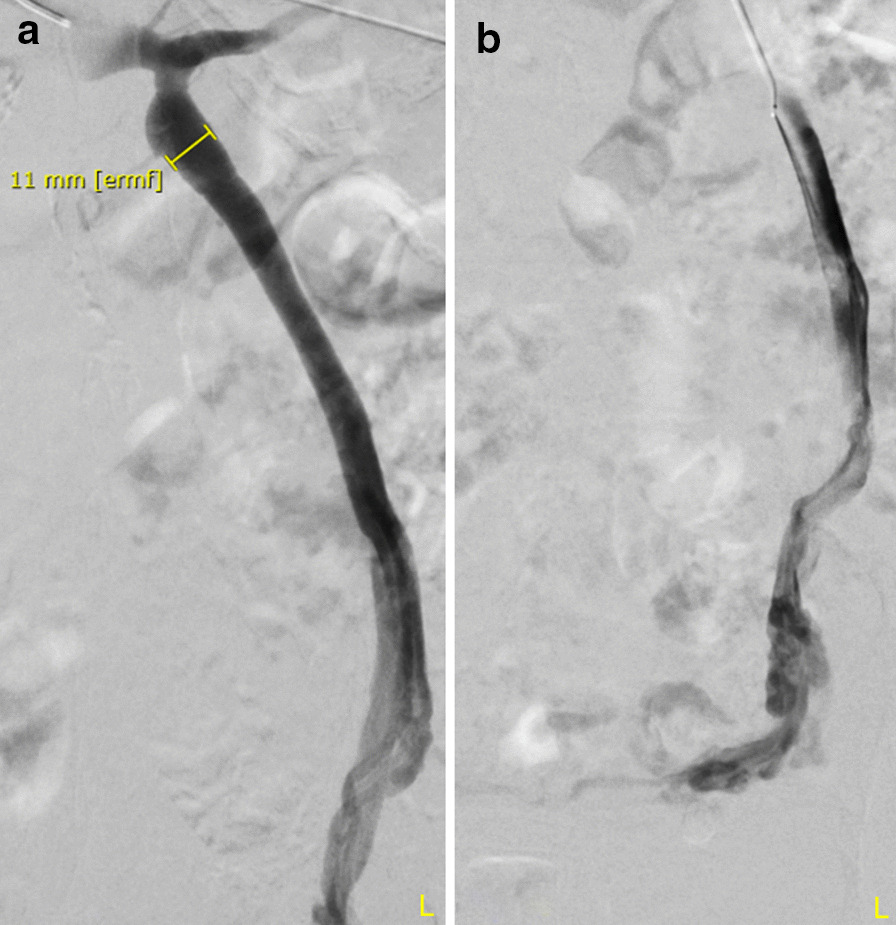
Angiography of left ovarian vein before coiling. **a** An insufficient left ovarian vein dilated up to 11 mm, **b** a prompt reverse flow to the small pelvis

**Fig. 2:**
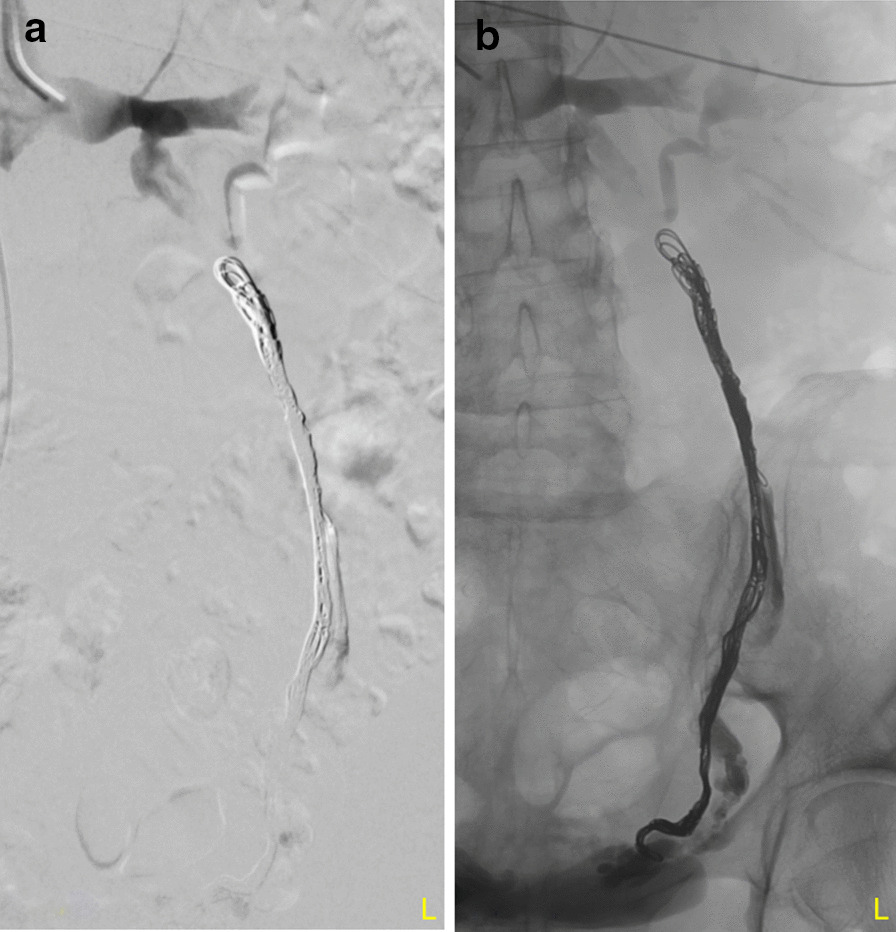
Angiography of left ovarian vein after coiling. **a** After coiling, there is no more flow depictable. **b** Position of the coils in the left ovarian vein

A 69-year old multipara with three vaginal deliveries and no preexisting medical conditions was admitted to our center due to chronic pelvic pain without morphologic correlates. The patient reported pelvic heaviness and diffuse pelvic pain, gradually increasing over recent years. The pain was described to characteristically increase during the course of the day and peak at a visual analog scale (VAS) score of 7 in the evenings, significantly impairing the patient’s quality of life over time. The patient could not identify an exact time point of symptom onset or a trigger of the pelvic pain. Gynecological examination showed no significant results, and laboratory tests showed no signs of inflammation. A diagnostic abdominal multiphase contrast CT scan revealed dilated uterine and ovarian veins (left ovarian vein up to 11 mm diameter, right 7 mm). No other pathologic findings were recorded. After ruling out any other causative pathology, PCS was suspected despite the patient’s menopausal state, based on both radiological findings and characteristic pain anamnesis. The patient declined conservative therapeutic approaches and consented to an interventional coil embolization of both ovarian veins after a particular discussion about the unusual suspected postmenopausal diagnosis. Embolization was performed by an experienced interventional radiologist in an outpatient setting within 3 weeks after the initial diagnosis. After administration of local anesthesia, the right femoral vein was cannulated, and a short 6-French sheath was introduced with a guide wire. A 5-French Sidewinder I angiographic catheter was placed for exploration of the left renal vein as previously described [17]. A diagnostic angiography performed during the procedure demonstrated a dilated left ovarian vein with reverse flow corresponding to venous insufficiency, as previously suspected (Fig. 1a). The contrast agent reached the small pelvis without resistance (Fig. 1b). After cannulation with a Penumbra Lantern^®^ Microcatheter (Alameda, CA, USA) and exploration with a Boston Scientific Fathom™ 16 guide wire (Marlborough, MA, USA), the left ovarian vein was sealed in its whole course with Penumbra Ruby^®^ Coils (Alameda, CA, USA) (Fig. 2b). No remaining reverse flow was demonstrated on diagnostic angiography (Fig. 2a). The procedure was repeated on the contralateral side. Similarly, the right ovarian vein, presenting as a regular anatomical variant ending in the inferior vena cava, was explored using a Sidewinder I catheter. Diagnostic angiography demonstrated venous insufficiency and reverse flow of the right ovarian vein. After sealing with two additional coils as previously described for the right ovarian vein, no reverse flow was observed. The procedure was performed without complications, and the patient left the hospital two hours after without pain or symptoms. At the follow-up visits three weeks and 18-months later, the patient reported no symptoms with a VAS score of 0, and no use of analgesics for pelvic pain.

## Discussion and conclusions

Even though no clinically confirmed case of postmenopausal PCS has been systematically reported to date, isolated references of postmenopausal patients who fulfill diagnostic criteria and experience a lasting alleviation of symptoms after respective standard treatment for PCS strongly suggest its incidence. The herein described case of an interdisciplinarily managed, postmenopausal PCS patient experiencing prompt alleviation of symptoms after interventional coil embolization thereby challenges the classic perception of a strictly premenopausal pathology.

A case report by Bildircin et al. [16] described a 44-year old multipara who was reported to have had PCS for 9 months. Therapeutic hysterectomy and bilateral salpingo-oophorectomy led to quick alleviation of symptoms, and intraoperatively, bilaterally dilated ovarian veins seemed to support the suspected diagnosis [16]. However, a histologically confirmed 4 × 5 cm mature teratoma of the right ovary was removed during the surgery, and no pain history was reported, which challenges the correct diagnosis of PCS in this specific case. Further, no assessment of the 44-year-old patient’s menopausal status was provided.

A study by Siqueira et al. included 22 symptomatic PCS patients up to age 55 who were treated by embolization of uterine varices. Of note, the authors reported that seven patients (31.8%) were postmenopausal upon inclusion but did not clarify how menopausal status had been assessed. No significant difference in therapy success was reported between pre- and postmenopausal patients [15]. Similarly, the largest retrospective study of endovascular treatment of PCS to date included 202 patients with a mean age of 43.5 years (range 27–57 years), and is also likely to have included postmenopausal patients [18]. The authors do not challenge the fact of menopausal status in their patient cohort, despite the prevailing literature documenting PCS as a premenopausal disease. Neither respective study, however, featured a gynecological patient cohort as patients were primarily screened for varicose veins of the lower extremities after examination by a vascular surgeon. No gynecological examination was performed to confirm the diagnosis of PCS or to question menopausal status. Moreover, no precise definition of diagnostic criteria for PCS was given, which may leave the correct diagnosis of PCS in question.

A recent exploratory study by Szaflarksi et al. evaluating the prevalence of ovarian venous congestion in adult patients may be considered of particular interest, as authors report 13.7% of 1042 female abdominal and pelvic CT scans to show venous congestion. The average age of the observed patient cohort was 47 years, indicating that a significant percentage of patients may have been of postmenopausal state. Due to the exploratory design and radiological focus, however, authors did not report whether or not patients fulfilled clinical criteria of PCS [19].

In the present case, the characteristic pain anamnesis and pre-interventionally described congested veins with reverse flow during angiography secured the diagnosis of postmenopausal PCS. Immediate alleviation of symptoms after the intervention supports that the congested veins induced the symptoms. Based on both previous literature and the present observation, it may be assumed menopausal status may not be an optimal diagnostic criterium to identify patients eligible for respective available treatment options. Moreover, postmenopausal PCS patients may be successfully treated following the same standardized procedures as previously described for premenopausal patients. This assumption, however, has to be confirmed in larger patient cohorts to allow for general applicability and to improve estimates of postmenopausal PCS prevalence and demographics.

Interventional coil-embolization of ovarian veins, as performed in the present case of a postmenopausal patient, may be considered a safe and effective gold standard to alleviate symptoms of venous congestion. A systematic review of 473 patients who underwent interventional coil embolization reported clinical alleviation of symptoms in 82.1–100% of cases. Complications were reported to be rare and comparably mild, such as local hematoma after cannulation. Recurrence rates were reported to be minimal [3]. Laborda et al. reported a remission of pain in 93.9% of patients with a follow-up of 5 years, with approximately one-third of patients achieving complete symptom relief. Thirteen percent experienced clinical recurrence of any degree. Four coil dislocations were described, which did not provoke subsequent complications [18]. Three more interventional-radiological trials including 31, 19, and 10 patients, respectively, reported comparable results and face comparable limitations to those previously discussed [20–22].

Both the interdisciplinary management of interventional radiologists and gynecologists as well as the long follow-up period represent particular strengths of the present report, which thereby provides the first systematically contextualized and documented observation of postmenopausal PCS. However, supplementary earlier imaging results to assess the time at which the congestion of pelvic veins occurred would have added value to the present case. A pre- and post-interventional standardized quality of life questionnaire might have added valuable quantifiable clinical information as a starting point for further clinical studies.

In contrast to the previous, particularly older literature, PCS does not appear to be solely limited to premenopausal patients. As patient history remains one of the main pillars of accurate diagnosis, introducing the concept of postmenopausal PCS to current research may greatly aid in standardizing therapeutic approaches for affected postmenopausal patients.

## Data Availability

Data and material are available on request from the corresponding author.

## Abbreviations

CT: Computed tomography
PCS: Pelvic congestion syndrome
VAS: Visual analog scale
